# Bacterial Isolates and Antimicrobial Susceptibility Profiles in Mares with Endometritis in Zhangye, China

**DOI:** 10.3390/ani16213531

**Published:** 2026-11-08

**Authors:** Xijun Zhang, Rui Ma, Qi Wang, Weitao Dong, Yong Zhang, Jie Yang

**Affiliations:** 1 College of Life Science and Engineering, Northwest Minzu University, Lanzhou 730000, China; 2 College of Veterinary Medicine, Gansu Agricultural University, Lanzhou 730000, China; 3 Gansu Key Laboratory of Animal Generational Physiology and Reproductive Regulation, Lanzhou 730000, China

**Keywords:** bacterial endometritis, pathogen isolation and identification, Zhangye, Gansu Province, China

## Abstract

Equine endometritis impairs fertility, but regional data remain scarce in Zhangye, Gansu Province, China, a major production base for equine serum-derived biological products. This cross-sectional study investigated endometritis and associated bacterial isolates in 985 mares from five intensive horse farms. Mares with reproductive abnormalities underwent clinical, ultrasonographic, and cytological evaluation. Laboratory investigations included culture of low-volume uterine flush samples, biochemical testing, 16S rRNA gene sequencing, phylogenetic analysis, and antimicrobial susceptibility testing. Among the 156 mares selected for further evaluation, 35 were diagnosed with endometritis. This corresponded to an apparent prevalence of 3.6% in the full surveyed population (35/985; 95% confidence interval, 2.5–4.9%) and a detection rate of 22.4% in the selected subgroup (35/156; 95% confidence interval, 16.2–29.8%). Clinical manifestations were generally subtle. Thirty culture-positive samples yielded 38 isolates across six genera. *Escherichia coli*, *Streptococcus equi* subsp. *zooepidemicus*, and *Staphylococcus aureus* predominated. A single bacterial taxon was recovered from 22 (73.3%) culture-positive mares, whereas multiple bacterial taxa were recovered from eight (26.7%). *Escherichia* and *Streptococcus* isolates showed relatively high resistance, whereas *Staphylococcus* and *Bacillus* isolates exhibited lower overall resistance. Limited geographical coverage, small isolate numbers, incomplete antimicrobial-use records, and unexamined resistance genes constrained interpretation. These findings support early screening of mares with reproductive disorders and antimicrobial selection guided by culture and susceptibility testing.

## Simple Summary

Endometritis, an inflammation of the uterine lining, can reduce fertility in mares. We examined 985 mares from five farms in Zhangye, China, a major center for biological products derived from horse blood. Endometritis was diagnosed in 35 mares (3.6%). Bacteria were recovered from 30 affected mares, with substantial differences in antibiotic resistance among bacterial groups. These findings highlight the importance of early examination of mares with reproductive problems and laboratory testing to guide antibiotic selection. Although limited to one region, this study provides information to support reproductive management and responsible antibiotic use.

## 1. Introduction

Gansu Province has long served as a major region for horse breeding and husbandry in northwestern China. Since the establishment of the Shandan Horse Farm during the Han Dynasty, the region has accumulated a long history of equine breeding, selection, and production. At present, supported by local equine genetic resources such as the Shandan horse and Hequ horse, Gansu has developed a relatively complete industrial chain for horse serum-derived biological products and has become a key supplier of equine-derived biological raw materials in China. Therefore, Gansu Province is of considerable strategic importance to the development of China’s equine industry.

Equine endometritis is a major cause of infertility in mares [1,2,3,4]. Its etiology is multifactorial and may involve microbial infection, mechanical injury, endocrine disorders, impaired uterine clearance, aging, malnutrition, and genetic factors [5]. Bacterial infection is an important contributing factor. Antimicrobial use is subject to product-specific restrictions in equine serum production; for example, the Chinese Pharmacopeia (2020 edition) prohibits penicillin, other β-lactam antibiotics, and streptomycin in horses used to produce immunoserum products for human use [6]. However, information on bacterial isolates associated with equine endometritis and their antimicrobial susceptibility remains limited in this region. This study therefore estimated the apparent prevalence of endometritis on five intensive horse farms in Zhangye, characterized aerobic bacterial isolates recovered from affected mares, and evaluated antimicrobial susceptibility in the predominant genera to inform local disease management and antimicrobial selection.

## 2. Materials and Methods

### 2.1. Animals

The survey was conducted during the local breeding season (April–October) in 2021 and 2022. Five intensive horse farms in Zhangye, Gansu Province, were selected to provide broad geographical coverage. Five intensive horse farms in Zhangye, Gansu Province, were selected to provide broad geographical coverage. Five intensive horse farms in Zhangye, Gansu Province, were selected to provide broad geographical coverage within the study area. The surveyed farms were of comparable scale, with 140–259 mares examined per farm (Supplementary Table S1). Each farm underwent a single cross-sectional screening, during which all eligible mares present were examined. The study population comprised 985 mares: Hequ horses (*n* = 577), Shandan horses (*n* = 186), Datong horses (*n* = 73), and crossbred mares or mares without recorded breed information (*n* = 149). Individual height at the withers and body weight were not available in the farm records used for this study and were not measured during the survey. Mares of any age and reproductive status were eligible. The farms practiced natural mating, with one or two stallions housed with groups of mares in each pen. Individual mating events were not systematically recorded, and the available reproductive histories did not permit reliable classification of mares as maiden, barren, or foaling. Farm-level breed and age distributions for the surveyed population, together with individual age and parity data for mares diagnosed with endometritis, are provided in Supplementary Table S1. Mares treated with an antimicrobial during the preceding 3 months were excluded. No mares with severe systemic disease or major reproductive-tract injury were identified. Ethical approval was granted by GSC-Eth-VMC2021-093.

### 2.2. Screening and Case Definition

All mares underwent physical and vaginal examination, review of reproductive history and transrectal ultrasonography. The definition of equine endometritis used in this study was based on previous reports [7]. A composite case definition was used to integrate complementary clinical, ultrasonographic, and cytological findings. For this study, endometritis was diagnosed when at least two of the following three criteria were present: (1) compatible clinical or reproductive-history findings; (2) intrauterine fluid detected by ultrasonography; and (3) cytological inflammation. The assessment methods and diagnostic criteria are summarized in Table 1.

### 2.3. Endometrial Cytology and LVF Sampling

Sampling was conducted during the local breeding season but was not scheduled according to individual estrous-cycle stage. The estrous-cycle stage at cytobrush sampling and subsequent LVF sampling was not specifically recorded. Isolation measures were implemented for mares with suspected reproductive problems during the sampling period to prevent natural mating. Endometrial cytology samples were obtained using a cytobrush passed through the cervix and applied at multiple sites in the uterine body and horns. The brush was rolled directly onto glass slides, which were stained with Diff-Quik. Three fields at 400× magnification were examined per slide, and the mean neutrophil count was calculated. A mean of ≥2 neutrophils per field was classified as cytological inflammation [8,9,10]. For mares diagnosed with endometritis, a low-volume uterine flush (LVF) sample was collected within 3 days of cytological sampling. No uterine lavage, hormonal treatment, or antimicrobial administration occurred during this interval. The tail was wrapped, and the vulva and perineal region were cleaned with benzalkonium bromide and rinsed with sterile saline. A sterile uterine catheter was introduced into the uterine body, 50–100 mL sterile physiological saline was infused, and approximately 30 mL was recovered. Samples were immediately refrigerated at 4 °C, transported to the laboratory on the day of collection and processed on arrival.

### 2.4. Aerobic Bacterial Culture and Identification

Each LVF sample was centrifuged at 500× *g* for 10 min. Most supernatant was discarded, and the pellet was resuspended in the remaining fluid to a final volume of approximately 2 mL. Three replicate 10% sheep blood agar plates were each inoculated with 20 µL and incubated aerobically at 37 °C for 36–48 h. Culture positivity was determined by experienced laboratory personnel based on the amount and consistency of colony growth across replicate plates; a prespecified quantitative colony-count threshold was not used. Distinct colony morphotypes were subcultured on 10% sheep blood agar for two to three passages until pure cultures were obtained. All isolates underwent Gram staining and biochemical testing, including methyl red-Voges-Proskauer, carbohydrate fermentation, indole, urease, coagulase, catalase, nitrate reduction and haemolysis tests as appropriate.

For molecular identification, each purified isolate was grown overnight in 4 mL nutrient broth at 37 °C with shaking at 180 rpm. Genomic DNA was extracted using a bacterial genomic DNA extraction kit (Tiangen Biochemical Technology Co., Ltd., Beijing, China). The 16S rRNA gene was amplified with primers 27F (5′-AGAGTTTGATCCTGGCTCAG-3′) and 1492R (5′-GGTTACCTTGTTACGACTT-3′). The 20 µL reaction contained 10 µL 2× Taq PCR Mix, 0.5 µL of each primer (10 µM), 1.5 µL DNA template and nuclease-free water. Cycling conditions were 94 °C for 5 min; 34 cycles of 94 °C for 30 s, 56 °C for 30 s and 72 °C for 1 min; and 72 °C for 5 min. Products of approximately 1500 bp were purified and sequenced commercially. Species assignments were based on the best BLAST match (generally >99% identity) together with phenotypic and biochemical findings. Maximum-likelihood phylogenetic trees were constructed separately for *Escherichia*, *Staphylococcus*, *Streptococcus*, and *Bacillus* using MEGA version 11.0. Within the genus *Streptococcus*, only the 6 *Streptococcus equi* subsp. *zooepidemicus* isolates were included in the phylogenetic analysis; the single *Streptococcus agalactiae* isolate was not included because only one isolate of this species was recovered.

### 2.5. Antimicrobial Susceptibility Testing

Antimicrobial susceptibility testing by the disk-diffusion method was performed on isolates from the four predominant genera, which collectively accounted for the majority of the recovered isolates: *Escherichia* spp. (*n* = 8), *Staphylococcus* spp. (*n* = 11), *Streptococcus* spp. (*n* = 7), and *Bacillus* spp. (*n* = 6). Genus-specific antimicrobial panels were selected according to local antimicrobial-use practices and their anticipated clinical relevance. Collectively, 20 antimicrobial agents were evaluated: the β-lactams penicillin G (PG, 10 U/disk), amoxicillin (AMX, 10 μg/disk), ceftazidime (CAZ, 30 μg/disk), and cefalotin (CF, 30 μg/disk); the aminoglycosides gentamicin (GEN, 10 μg/disk), streptomycin (SM, 10 μg/disk), and kanamycin (KAN, 30 μg/disk); the fluoroquinolones norfloxacin (NOR, 10 μg/disk), ofloxacin (OFL, 5 μg/disk), and ciprofloxacin (CIP, 5 μg/disk); the macrolides clarithromycin (CLR, 15 μg/disk) and erythromycin (ERY, 15 μg/disk); the glycopeptide vancomycin (VAN, 30 μg/disk); polymyxin B (PB, 300 U/disk); the tetracyclines tetracycline (TET, 30 μg/disk) and doxycycline (DOX, 30 μg/disk); the folate-pathway inhibitors trimethoprim (TMP, 5 μg/disk), sulfisoxazole (SIZ, 300 μg/disk), and trimethoprim–sulfamethoxazole (TMP–SMX, 1.25/23.75 μg/disk); and the rifamycin rifampicin (RIF, 5 μg/disk).

Bacterial suspensions were adjusted to a turbidity equivalent to a 0.5 McFarland standard and uniformly inoculated onto Mueller–Hinton agar. Antimicrobial disks purchased from Hangzhou Binhe Microbial Reagent Co., Ltd. (Hangzhou, China) were placed on the inoculated agar surface, and the plates were incubated at 35–37 °C for 18–24 h. Inhibition-zone diameters were measured along two perpendicular axes using a ruler or caliper, and the mean diameter was recorded in millimeters. Each isolate was individually classified as susceptible, intermediate, or resistant according to the organism- and antimicrobial-specific interpretive criteria provided by the manufacturer. Tests yielding equivocal or anomalous results were repeated. The genus-specific antimicrobial panels, disk potencies, and corresponding interpretive criteria are presented in Supplementary Table S2.

### 2.6. Statistical Analysis

Data were collected and processed using IBM SPSS Statistics version 27.0 and Microsoft Excel 2010. Apparent prevalence and detection rates were calculated as proportions with exact binomial 95% confidence intervals (CIs). Differences in prevalence among farms and age groups were analyzed using the chi-square test, and pairwise comparisons were adjusted using the Bonferroni correction. The response of each bacterial isolate to each antimicrobial agent was classified as resistant, intermediate, or susceptible. Antimicrobial susceptibility results were summarized descriptively as the numbers and proportions of resistant, intermediate, and susceptible isolates for each genus–antimicrobial combination. Pairwise comparisons were adjusted using the Bonferroni correction. A value of *p* < 0.05 was considered statistically significant.

## 3. Results

### 3.1. Distribution of Mares with Endometritis

**Figure 1:**
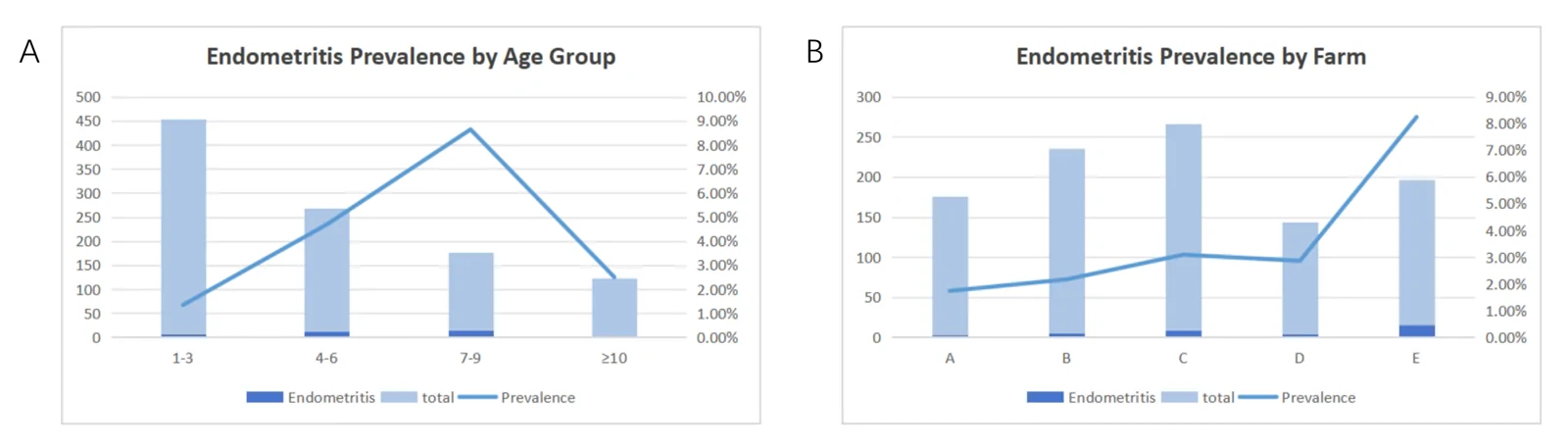
Apparent prevalence of endometritis in mares by age group (**A**) and farm (**B**). In each panel, the bars represent the numbers of mares examined and mares diagnosed with endometritis (left y-axis), while the line indicates the apparent prevalence of endometritis within each group (right y-axis, %). (**A**), age-group analysis; (**B**), farm-level analysis.

Among the 985 mares examined across five intensive horse farms (A–E) in Zhangye, Gansu Province, 35 were diagnosed with endometritis, yielding an apparent prevalence of 3.6% (95% CI, 2.5–4.9%). Among the 35 mares diagnosed with endometritis, age ranged from 3 to 11 years and parity ranged from 1 to 9. Supplementary Table S1 presents farm-level information on the surveyed population, including breed and age distributions, together with individual age, parity, and bacterial isolation results for the affected mares. The prevalence differed significantly among farms (χ^2^ = 15.2, df = 4, *p* = 0.004), ranging from 1.7% (3/173) on Farm A to 8.2% (15/182) on Farm E. A significant age-related pattern was also observed (χ^2^ = 20.0, df = 3, *p* < 0.001), with prevalence increasing from 1.3% (6/447) in mares aged 1–3 years to 4.7% (12/256) in those aged 4–6 years and peaking at 8.6% (14/162) in mares aged 7–9 years. Although the prevalence declined to 2.5% (3/120) in mares aged ≥10 years, mares aged 7–9 years had a significantly higher prevalence than those aged 1–3 years after Bonferroni adjustment (adjusted *p* < 0.001) (Figure 1).

### 3.2. Bacterial Identification

Among the 35 LVF samples included in this study, bacteria were successfully isolated from 30 samples. A total of 38 bacterial isolates were purified. These isolates belonged to six genera, including *Staphylococcus* spp. (11/38, 28.9%), *Escherichia* spp. (8/38, 21.1%), *Streptococcus* spp. (7/38, 18.4%), *Bacillus* spp. (6/38, 15.8%), *Klebsiella* spp. (4/38, 10.5%), and *Actinomyces* spp. (2/38, 5.3%). Specifically, the isolates included eight *Escherichia coli* isolates, six *Streptococcus equi* subsp. *zooepidemicus* isolates, six *Staphylococcus aureus* isolates, five other *Staphylococcus* spp. isolates, two *Bacillus cereus* isolates, three *Bacillus pumilus* isolates, one *Bacillus atrophaeus* isolate, four *Klebsiella pneumoniae* isolates, two *Actinomyces* spp. isolates, and one *Streptococcus agalactiae* isolate.

**Figure 2:**
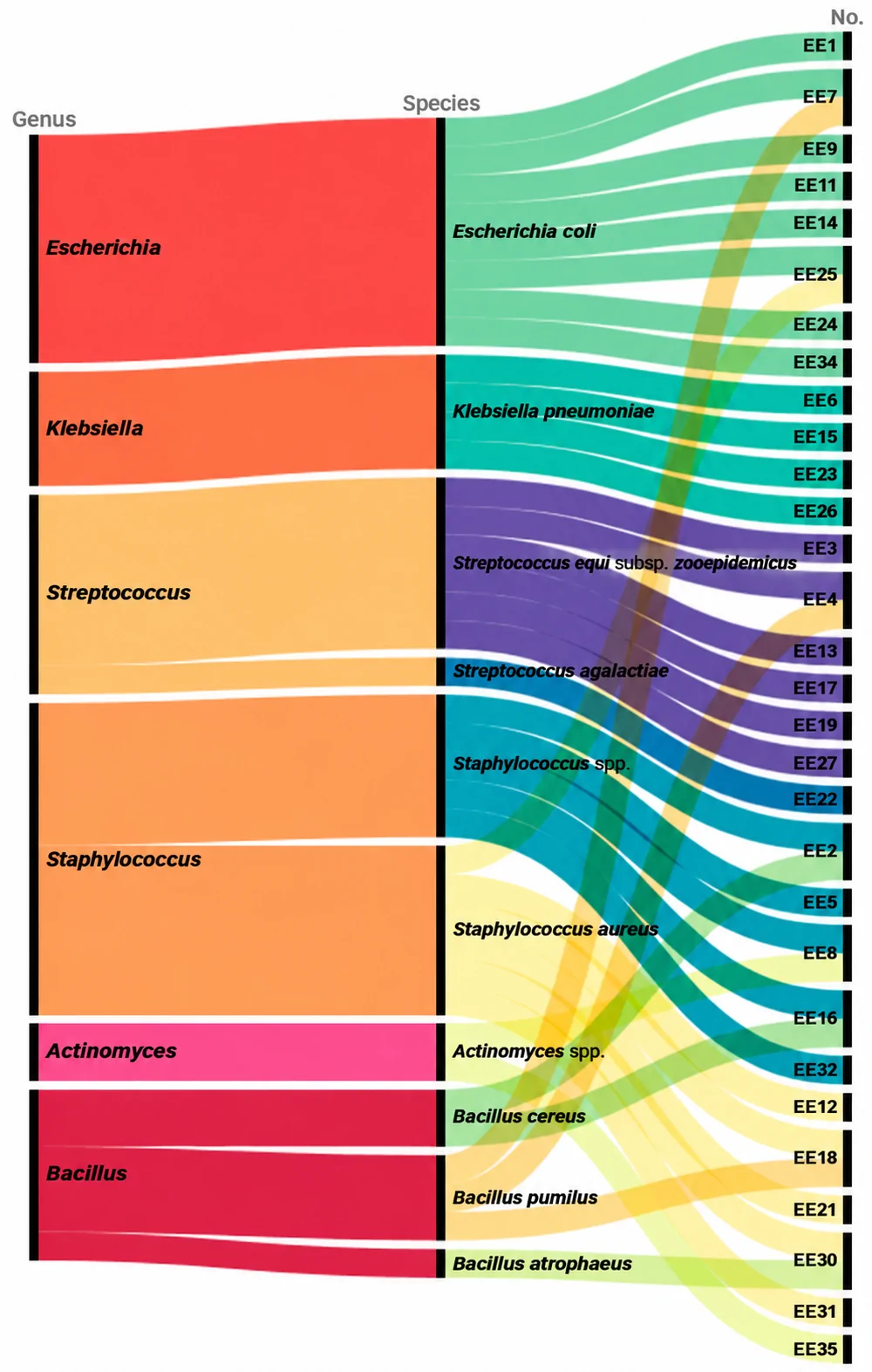
Sankey diagram showing the distribution of bacterial isolates by genus, species, and sample identifier.

**Figure 3:**
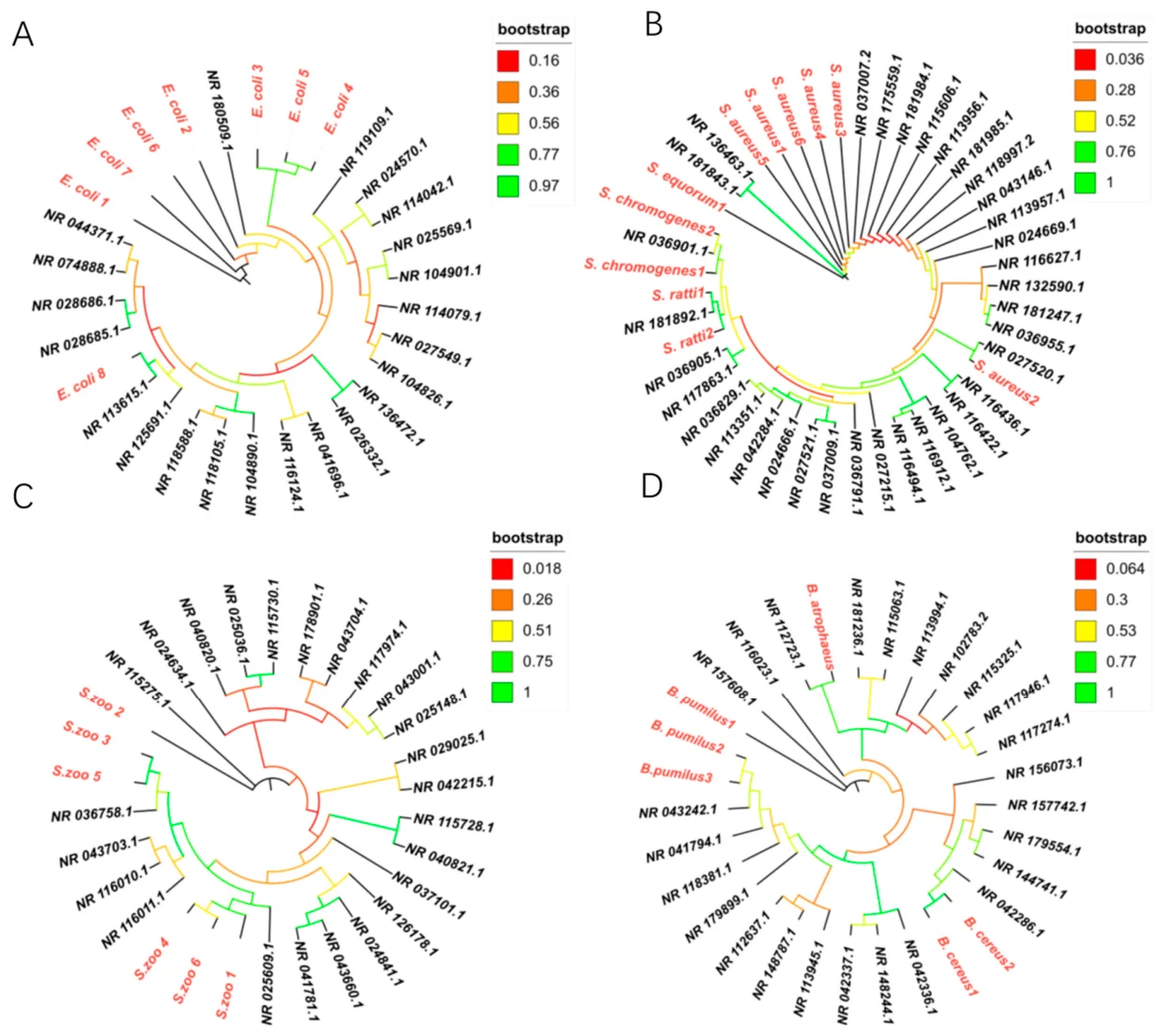
Maximum-likelihood phylogenetic trees of *Escherichia*, *Staphylococcus*, *Streptococcus*, and *Bacillus* isolates. (**A**): *E. coli*; (**B**): *Staphylococcus* spp.; (**C**): *S. equi* subsp. *zooepidemicus*; (**D**): *Bacillus* spp. Red labels indicate isolates obtained in this study, whereas black labels indicate reference sequences.

Among the 30 culture-positive mares, a single bacterial taxon was recovered from 22 samples (73.3%), whereas multiple bacterial taxa were recovered from eight samples (26.7%) (Figure 2). Phylogenetic trees were constructed for the predominant bacterial genera, including *Escherichia*, *Staphylococcus*, *Streptococcus*, and *Bacillus*, using MEGA software version 11.0 (Figure 3). Within the genus *Streptococcus*, only the six *Streptococcus equi* subsp. *zooepidemicus* isolates were included in the phylogenetic analysis, because only one *Streptococcus agalactiae* isolate was recovered. The results showed that the isolated strains were closely related within their respective genera.

### 3.3. Antimicrobial Susceptibility

**Figure 4:**
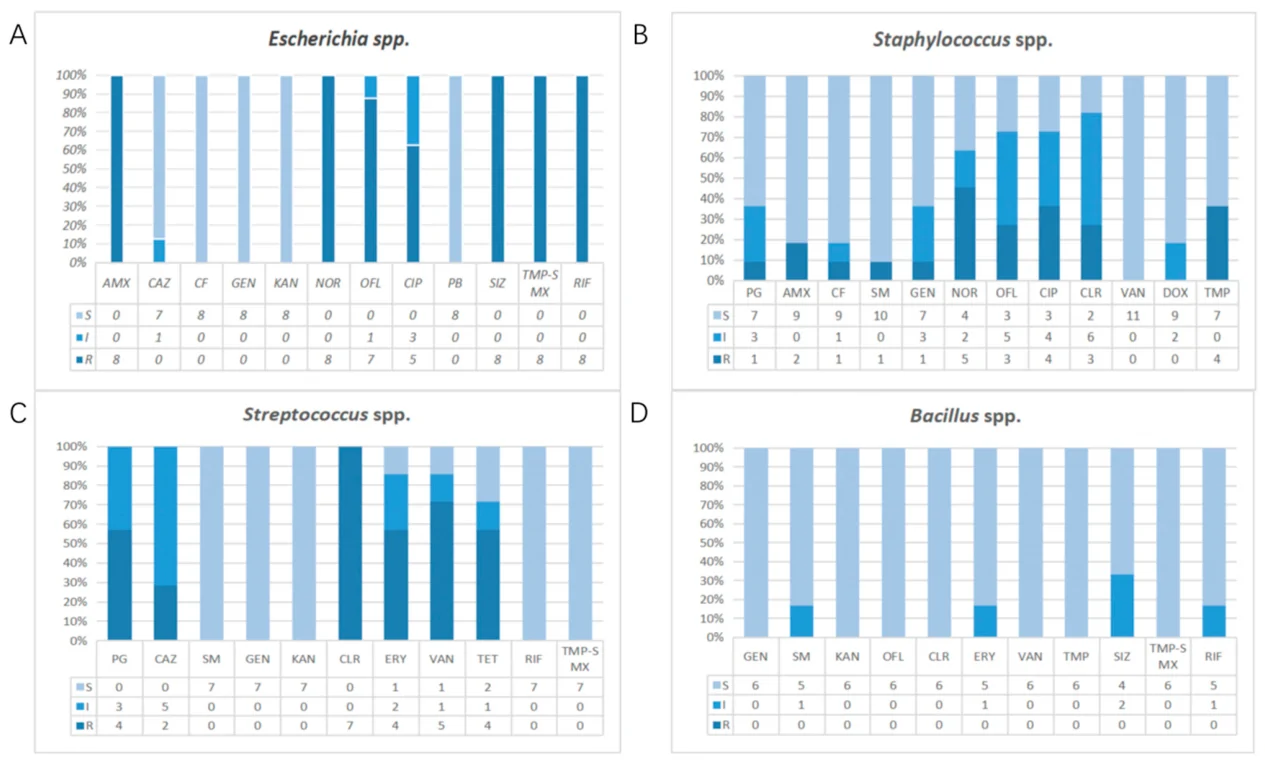
Antimicrobial susceptibility profiles of *Escherichia* (**A**), *Staphylococcus* (**B**), *Streptococcus* (**C**), and *Bacillus* (**D**) isolates. The bars indicate the percentages of isolates classified as susceptible (S), intermediate (I), or resistant (R) to each antimicrobial agent. The corresponding numbers of isolates in each category are provided in the table beneath each panel. S, susceptible; I, intermediate; R, resistant.

The antimicrobial susceptibility profiles differed markedly among the four bacterial genera. All *Escherichia* spp. isolates (*n* = 8) were susceptible to CF, GEN, KAN, and PB (8/8), whereas all isolates were resistant to AMX, NOR, SIZ, TMP–SMX, and RIF (8/8). High resistance was also observed for OFL (7/8) and CIP (5/8), indicating a broad resistance profile (Figure 4A). Among *Staphylococcus* spp. isolates (*n* = 11), all isolates were susceptible to VAN (11/11), while high susceptibility was observed for SM, AMX, CF, and DOX (9/11–10/11). Resistance was most frequently detected against NOR (5/11), followed by CIP and TMP (4/11 each). Relatively high proportions of isolates were categorized as intermediate to CLR, OFL, and CIP (Figure 4B). All *Streptococcus* spp. isolates (*n* = 7) were susceptible to SM, GEN, KAN, RIF, and TMP–SMX (7/7) but resistant to CLR (7/7). Resistance to VAN was detected in 5/7 isolates, whereas PG, ERY, and TET each showed resistance in 4/7 isolates. In addition, most isolates showed intermediate susceptibility to CAZ (5/7) (Figure 4C). No resistance to the tested antimicrobial agents was detected among the six *Bacillus* spp. isolates (0/6). All six isolates were susceptible to most tested antimicrobial agents (6/6), whereas four or five isolates were susceptible to SIZ, SM, ERY, and RIF (4/6–5/6; Figure 4D). The susceptibility patterns of *Staphylococcus* spp. and *Streptococcus* spp. were more dependent on the antimicrobial agent tested.

## 4. Discussion

In Zhangye, Gansu Province, mare breeding activities are mainly concentrated from April to October each year. In the present study, five intensive horse farms of comparable scale were investigated during this period. A total of 985 mares were examined, among which 35 were diagnosed with endometritis, corresponding to an apparent prevalence of 3.6%. Among the 156 mares with reproductive problems recorded within the previous year, including failure to conceive, repeated estrus, or abnormal vulvar discharge, the detection rate of endometritis was 22.4%. These findings indicate that although the overall apparent prevalence of endometritis was relatively low among all mares on the farms, the disease remained common in mares with reproductive disorders. The number of cases varied markedly among farms, with Farm E having the highest number of cases. The number of cases varied markedly among farms, with Farm E having the highest number of cases. However, the reasons for these differences remain unclear, as farm-level management practices and other potential contributing factors were not systematically assessed. However, these factors were not formally assessed. These observations suggest that farm management may influence both the opportunity for bacteria to enter the reproductive tract and the timely detection of cases with subtle clinical manifestations. However, endometrial cytology was performed only in mares that met the initial screening criteria. Consequently, subclinical cases among mares not selected for further evaluation may have been missed, potentially affecting the apparent prevalence estimates and comparisons among farms and age groups. These comparisons should therefore be interpreted cautiously and not as evidence of independent risk factors.

Some intensive horse farms in Gansu Province are mainly used for the production of equine-derived serum products. Under this production system, production mares are replaced relatively frequently, and older mares with reduced reproductive performance are more likely to be removed from production or culled. In the present study, the prevalence of endometritis increased across the three age groups below 10 years but declined in the oldest age group. This apparent decrease should be interpreted cautiously, as it may partly reflect the reduced number of older mares retained on the farms rather than a true decrease in disease risk. Increases in age and parity may be accompanied by gradual changes in endocrine status, metabolic profiles [11], perineal conformation, vulvar closure function, uterine position, and myometrial contractility, which may weaken uterine clearance of inflammatory exudates and pathogenic microorganisms [12]. Notably, most affected mares in this study lacked obvious fever or large amounts of abnormal vulvar discharge. Instead, they mainly presented with failure to conceive, repeated estrus, intrauterine fluid accumulation, and cytological evidence of inflammation. This suggests that many cases in local horse farms had relatively subtle or nonspecific clinical manifestations. Therefore, screening based solely on obvious clinical signs may lead to missed diagnoses.

In the present study, 30 of the 35 low-volume uterine flush samples were positive for bacterial culture, and a total of 38 bacterial isolates were obtained. The five culture-negative cases may reflect fungal or anaerobic infections, although these were not specifically investigated in this study. At the species level, *Escherichia coli* was the most frequently detected bacterium, followed by *Streptococcus equi* subsp. *zooepidemicus* and *Staphylococcus aureus.* These bacteria were the predominant aerobic bacterial isolates recovered from mares diagnosed with endometritis in the surveyed population. The major bacterial species identified in this study were generally consistent with those reported in previous studies from China and other countries [8,13,14,15,16,17]. However, the relative proportions of different bacterial species varied among studies. Such variation may be related to regional climate, breeding environment, mare breed, reproductive management practices, antimicrobial use patterns, and differences in sampling and bacterial identification methods. Although isolation measures were implemented during the sampling period, the preceding cytobrush procedure could have introduced bacteria and influenced subsequent LVF culture results. The absence of recorded estrous-cycle stages prevented assessment of this potential influence in relation to cycle stage. Therefore, the recovered isolates should not all be assumed to have caused the pre-existing inflammation.

Among the common pathogenic bacteria identified in this region, *Escherichia coli* is mainly derived from the intestinal tract and fecally contaminated environments. It may ascend into the uterus through the perineal and vulvar regions. Some *E. coli* strains possess strong adhesion capacity [18] and biofilm-forming ability [19,20], which may facilitate persistent infection when uterine clearance is impaired. *Streptococcus equi* subsp. *zooepidemicus* is a common opportunistic pathogen associated with equine endometritis [21,22]. When the protective barriers of the vulva, vagina, and cervix are compromised, or when uterine clearance is reduced, this organism may colonize the uterus and induce persistent inflammation [23,24]. *Staphylococcus aureus* and other *Staphylococcus* spp. are widely present on animal skin, mucosal surfaces [25,26], and in farm environments [27]. *Staphylococcus* spp. are common pathogens associated with equine endometritis [28]; their detection may be related to breeding, artificial insemination, postpartum examination, and vulvar hygiene conditions [29]. Although *Klebsiella pneumoniae* was isolated less frequently in this study, it is also an important pathogen associated with equine endometritis [28,30]. Its capsule and biofilm-forming ability [31] may contribute to prolonged infection and increased difficulty in treatment. *Bacillus* spp. are commonly found in soil, dust, and farm environments [32]. Their detection in the uterus may reflect opportunistic infection, but sampling contamination cannot be completely excluded. Therefore, interpretation should consider bacterial growth abundance, whether the isolate was predominant in culture, the degree of cytological inflammation, and repeat culture results.

Among the culture-positive mares, 73.3% had single-pathogen infections, whereas 26.7% had mixed infection. This suggests that single-pathogen infection was predominant in local cases, although mixed infections may increase treatment difficulty by altering the local uterine microenvironment and promoting biofilm formation [33,34]. Phylogenetic analysis showed that the selected isolates clustered with closely related reference sequences within their respective genera, supporting their taxonomic placement at the genus level. The isolates included in each tree showed relatively limited sequence divergence at the 16S rRNA gene level.

Antimicrobial susceptibility profiles differed among bacterial genera. *Escherichia* spp. showed complete resistance to AMX, NOR, SIZ, TMP–SMX, and RIF (8/8 for each agent), with high resistance to OFL (7/8) and CIP (5/8), whereas most isolates remained susceptible to CAZ, and all isolates were susceptible to CF, GEN, KAN, and PB (8/8 for each agent). *Streptococcus* spp. showed complete resistance to CLR (7/7) and relatively high resistance to VAN (5/7), PG (4/7), ERY (4/7), and TET (4/7), but remained susceptible to the aminoglycosides SM, GEN, and KAN, as well as RIF and TMP–SMX (7/7 for each agent). *Staphylococcus* spp. exhibited lower overall resistance, whereas no resistant *Bacillus* spp. isolates were detected (0/6). Repeated or empirical use of the same antimicrobial classes, together with inappropriate dosages or treatment durations, may impose selective pressure and promote the persistence of resistant strains [35]. However, because historical antimicrobial-use records and resistance determinants were not systematically investigated, the mechanisms underlying the observed resistance patterns remain uncertain. Therefore, antimicrobial treatment should, whenever possible, be guided by bacterial culture and susceptibility testing rather than prolonged empirical use of broad-spectrum agents.

Overall, this study preliminarily revealed the regional characteristics of equine bacterial endometritis in Zhangye, Gansu Province, in terms of epidemiological features, bacterial pathogen composition, and antimicrobial resistance profiles. These findings provide a basis for local horse farms to identify chronic and subclinical cases, perform etiological diagnosis, and select antimicrobial agents rationally. In particular, for intensive horse farms mainly engaged in the production of equine-derived serum products, strengthening reproductive management and standardizing antimicrobial use are important for maintaining mare reproductive performance, reducing the risk of antimicrobial resistance, and ensuring the quality of serum-derived raw materials. Owing to the limited survey area and number of bacterial isolates, the applicability of these findings to broader regions and different farming systems requires further validation. The lack of reliable reproductive-status classification prevented separate analysis of maiden, barren, and foaling mares. Whether breed characteristics contributed to the relatively low apparent prevalence also remains uncertain, as breed-specific effects were not evaluated.

## 5. Conclusions

This study investigated 985 mares from five intensive horse farms in Zhangye, Gansu Province, and identified 35 mares with endometritis, corresponding to an apparent prevalence of 3.6% (95% CI, 2.5–4.9%). Most affected mares presented with chronic or subclinical reproductive abnormalities, including failure to conceive, repeated estrus, intrauterine fluid accumulation, and cytological evidence of inflammation. Among the 35 low-volume uterine flush samples, 30 were positive for bacterial culture, and a total of 38 bacterial isolates were obtained. *Escherichia coli*, *Streptococcus equi* subsp. *zooepidemicus*, and *Staphylococcus aureus* were the predominant bacterial species detected. Recovery of a single bacterial taxon was the predominant culture pattern, although multiple taxa were recovered from some samples. Antimicrobial susceptibility testing revealed marked differences in resistance profiles among bacterial genera. *Escherichia* spp. and *Streptococcus* spp. showed relatively high levels of antimicrobial resistance, whereas *Staphylococcus* spp. exhibited a lower overall resistance level, and no resistant isolates were detected among *Bacillus* spp. In summary, among the mares examined on the five surveyed intensive horse farms in Zhangye, Gansu Province, endometritis was characterized by relatively subtle clinical manifestations, diverse associated bacterial isolates, and varying antimicrobial resistance profiles among the major bacterial genera. Therefore, early screening of mares with reproductive disorders should be emphasized in clinical practice, and antimicrobial agents should be selected rationally based on bacterial culture and antimicrobial susceptibility testing.

## Figures and Tables

**Table 1:** Assessment methods and diagnostic criteria for endometritis in mares.

Assessment Component	Assessment Method	Diagnostic Criterion
Clinical and reproductive-history findings	Review of reproductive history and physical and vaginal examinations	Compatible findings, including failure to conceive, repeated estrus, abnormal vulvar discharge, or an abnormal vaginal examination
Intrauterine fluid	Transrectal ultrasonography	Presence of intrauterine fluid
Endometrial cytology	Examination of three fields at 400× magnification and calculation of the mean neutrophil count	Mean neutrophil count ≥ 2 per field [8,9,10]

## Data Availability

The original contributions presented in this study are included in the article/Supplementary Material. Further inquiries can be directed to the corresponding authors.

## Supplementary Materials

The following supporting information can be downloaded at: https://www.mdpi.com/article/10.3390/ani16213531/s1, Table S1: Farm and mare characteristics and bacterial isolation results; Table S2: Antimicrobial susceptibility results and interpretive criteria.
